# Pathophysiological trajectories and biological consequences of early life trauma

**DOI:** 10.1080/20008198.2017.1351159

**Published:** 2017-09-29

**Authors:** Agorastos Agorastos

**Affiliations:** ^a^ Department of Psychiatry and Psychotherapy, University Medical Center Hamburg-Eppendorf, Hamburg, Germany

**Keywords:** Early life trauma, pathophysiological trajectories, biological consequences

## Abstract

Experience of early life stress (ELS) (childhood trauma, maltreatment, neglect, separation, physical and sexual abuse, parental loss and other severe anxiety/stress symptoms) is highly prevalent in the general population and constitutes a major public health problem (Gilbert et al., 2009). The objective of this paper is to review evidence on epidemiological risk factors, pathophysiological trajectories and neurobiological pathways exerting the enduring adverse effects of ELS and leading to compromised overall health in adulthood.

Prolonged neuropsychobiological alterations as sequelae of early trauma could mediate the risk of disease in adulthood, and lead to cumulative disadvantages and increased physical and mental morbidity in later life. In particular, a higher risk of psychiatric disorders (e.g. depression, post-traumatic stress disorder and other anxiety disorders) and their unfavourable outcomes (e.g. treatment resistance, suicide) has been associated with a history of ELS in several retrospective but also prospective studies, while the moderating interaction of ELS and pre-existing genetic vulnerability in these disorders has been extensively discussed in recent literature. Nevertheless, the chronic physical health consequences of childhood adversities may be as substantial as mental health consequences. Prior research suggests an association of ELS with cardiovascular, pulmonary and metabolic diseases, chronic inflammatory and pain syndromes, frequency of medical consultations and number of medical diagnoses. In addition, risk behaviour patterns such as substance use, and especially tobacco and alcohol consumption, are considered significantly increased in individuals with experience of ELS. Consequently, many studies have reported a negative impact of ELS on adult general mental and physical health-related quality of life, while recognizing the additive effect of the number or different types of childhood adversities is important for understanding their cumulative effect on later life adjustment (Agorastos, Pittman et al., 2014).

The distinct impact of ELS may lie in enhanced plasticity mechanisms during this period that lead to persistent functional and epigenetic alterations and to higher allostatic load over time. Experience of ELS has been shown to lead to an increased neuroendocrine stress response and vulnerability to stress, hypothalamic–pituitary–adrenal axis dysregulation, long-lasting alterations in emotional and psychophysiological reactivity, impaired adaptive functioning, malfunction of fear response circuits and structural changes in the central nervous system. ELS can also lead to epigenetic modifications in response to environmental influences through stress-related gene expression and, thus, play a central role in the long-term biological trajectories leading to stress-related disease, and may explain interindividual variation. In addition, ELS represents an independent risk factor for neuroendocrine–immunological abnormalities with the development, to some level, of a proinflammatory phenotype in adulthood. Finally, recent literature has focused on the potentially fundamental role of sleep and circadian rhythms in the development of ELS-related disorders. Central and peripheral circadian disruption after severe stress in childhood could engrave pathophysiological trajectories of ELS through impaired homeostatic balance, with overall neuroendocrine, immune, metabolic and autonomic dysregulation (Agorastos, Kellner et al., 2014).

Taken together, persistent structural, functional and epigenetic changes in neural circuits after ELS could foster chronic chronodisruption and thus mediate the risk of disease and resilience in adults, following a distinct dose–response relationship. The long-term effects of ELS may be conceptualized as a common developmental risk factor triggering a health-related risk cascade with high public health impact. Future studies should focus on prospective investigation of potential predictors and mediators, their temporal sequence and combined effects at epidemiological, biological and epigenetic levels, while taking into account the potentially delayed time-frame for the expression of their effects. Screening strategies for ELS need to be improved, as they would help to identify an individual’s risk level for disease development and predict the response to treatment, towards a better understanding of the relationship between gene and environmental exposures that impacts resilience.

## References

[CIT0001] Agorastos A., Kellner M., Baker D. G., & Otte C. (2014). When time stands still. An integrative review on the role of chronodisruption in PTSD. *Current Opinion in Psychiatry*, 27, 1–2. doi:10.1097/YCO.0000000000000079 25023884

[CIT0002] Agorastos A., Pittman J. O., Angkaw A. C., Nievergelt C. M., Hansen C. J., Aversa L. H., … Baker D. G. (2014). The cumulative effect of multiple childhood trauma types on self-reported symptoms of adult depression, PTSD, substance abuse and health-related quality of life in a large active-duty military cohort. *Journal of Psychiatric Research*, 58, 46–54. doi:10.1016/j.jpsychires.2014.07.014 25139009

[CIT0003] Gilbert R., Widom C. S., Browne K., Fergusson D., Webb E., & Janson S. (2009). Burden and consequences of child maltreatment in high-income countries. *Lancet*, 373(9657), 68–81. doi:10.1016/S0140-6736(08)61706-7 19056114

